# Can the nucleic acid Ct value of discharged patients infected with SARS-CoV-2 Omicron variant be 35?——A retrospective study on fluctuation of nucleic acid Ct values in SNIEC mobile cabin hospital

**DOI:** 10.3389/fcimb.2022.1059880

**Published:** 2022-12-19

**Authors:** Xu Zhuang, Yu Zheng, Shun Wei, Wei Zhai, Qixiang Song, Min Chen, Qingrong Xu, Yiling Fan, Junhua Zheng

**Affiliations:** ^1^ Department of Obstetrics and Gynecology, Renji Hospital, School of Medicine, Shanghai Jiao Tong University, Shanghai, China; ^2^ Department of Pulmonology, Renji Hospital, School of Medicine, Shanghai Jiao Tong University, Shanghai, China; ^3^ Department of Information Center, Renji Hospital, School of Medicine, Shanghai Jiao Tong University, Shanghai, China; ^4^ Department of Urology, Renji Hospital, School of Medicine, Shanghai Jiao Tong University, Shanghai, China; ^5^ Nursing Department, Renji Hospital, School of Medicine, Shanghai Jiao Tong University, Shanghai, China; ^6^ Department of Orthopaedics, Renji Hospital, School of Medicine, Shanghai Jiao Tong University, Shanghai, China; ^7^ Department of Neurosurgery, Renji Hospital, School of Medicine, Shanghai Jiao Tong University, Shanghai, China

**Keywords:** COVID-19, cycle threshold value, fluctuation, mobile cabin hospital, omicron

## Abstract

**Objective:**

To explore the meaning of cycle threshold (Ct) value fluctuation and the appropriateness of setting the discharge Ct value to 35, which is the current standard in Chinese guidelines.

**Method:**

A retrospective study was conducted on 95 patients with Ct value fluctuation (Ct value below 35 on day 3; group A) and 97 patients with a normal discharge process (control; group B). Their clinical characteristics and follow-up data were collected.

**Results:**

(1) There was no significant difference between the groups in age, gender distribution, number of vaccinations, initial ORF-Ct value, and initial N-Ct value. The proportion of patients complicated with chronic internal disorders, respiratory symptoms, and abnormal chest radiology in group A was significantly higher than that in group B. (2) Between the two groups, there was no significant difference in the ORF-Ct or N-Ct value on day 1, but the ORF-Ct and N-Ct values of group B on days 2 to 4 were significantly higher than those of group A. (3) There was no significant difference between the groups in the ORF-Ct value at discharge, but there was a significant difference in the N-Ct value at discharge. Seven days after discharge, almost 100% of the patients had been cured. The mean negative conversion interval of nucleic acid of the patients in group A was 14.5 ± 4.6 days, which was longer than that of the patients in group B (11.8 ± 4 days). (4) Logistic regression analysis showed that the ORF-Ct value on day 2 was the key factor influencing the Ct value fluctuation.

**Conclusion:**

The fluctuation of Ct value is only a normal phenomenon in the recovery period of the disease, and there is no need for excessive intervention. It is reasonable to set the Ct value of the discharge standard to 35 and retest the nucleic acid on the 10^th^ day after discharge for patients with underlying diseases or symptoms.

## 1 Introduction

In 2022, a wave of infection of the SARS-CoV-2 Omicron variant rapidly spread in Shanghai, China. According to recent literature, over six hundred thousand cases have been identified, nearly 90% of which are asymptomatic (Zhang et al., 2022). The Shanghai New International Expo Center (SNIEC) was the first large venue converted into a mobile cabin hospital and has admitted over ten thousand patients. Facing the epidemic outbreak, mobile cabin hospitals, as temporary isolation points, can only conduct daily nucleic acid tests on patients (National Health Commission of the People’s Republic of China, 2022).

According to the discharge criteria of novel coronavirus pneumonia in Shanghai, a patient can be released from isolation when their nucleic acid cycle threshold (Ct) values are at least 35 in two consecutive tests (National Health Commission of the People’s Republic of China,, 2022). However, the Ct values of patients who have already met the discharge criterion sometimes fluctuate on the day of discharge, resulting in delays in discharge. Therefore, further study should be conducted to determine the meaning of such fluctuation and the appropriateness of setting the discharge Ct value to 35.

## 2 Materials and methods

### 2.1 Materials

This retrospective study was approved by the ethics committee of Renji Hospital in accordance with the principle of informed consent and was conducted from May 8 to May 25. The clinical characteristics and nucleic acid Ct values of 192 patients were collected; 95 of these patients had nucleic acid Ct value fluctuation (group A), and the 97 remaining patients had a normal discharge process and were regarded as the control (group B). All patients were followed up on the seventh day after discharge.

### 2.2 Definitions

#### 2.2.1 Discharge criteria in China

According to the Chinese guidelines on the Diagnosis and Treatment Protocol for Novel Coronavirus Pneumonia (ninth version), patients who meet the following conditions can be discharged: (1) The body temperature has been normal for more than three days. (2) The respiratory symptoms have significantly improved. (3) Pulmonary imaging shows a significant improvement in acute exudative lesions. (4) The Ct values of the N and ORF genes (N-Ct and ORF-Ct, respectively) are at least 35 in two consecutive novel coronavirus nucleic acid tests with a 24 h sampling interval (National Health Commission of the People’s Republic of China, 2022).

#### 2.2.2 Time points

Day 1 was defined as the first of two consecutive days where the N-Ct and ORF-Ct values were at least 35. Each day after day 1 was set as day 2, day 3, and so on.

#### 2.2.3 Fluctuation of nucleic acid Ct value

The N-Ct or ORF-Ct value, detected *via* a novel coronavirus nucleic acid test, was below 35 on day 3, resulting in a delay in discharge.

#### 2.2.4 Nucleic acid Ct value criteria for negative

According to the Chinese guidelines on the Diagnosis and Treatment Protocol for Novel Coronavirus Pneumonia (ninth version), the Ct value criteria for negative conversion of nucleic acid was defined as the N-Ct and ORF-Ct value being at least 40. The time interval from the point of onset to the point of negative conversion of nucleic acid was the negative conversion interval of nucleic acid.

### 2.3 Statistical analysis

Statistical analysis was conducted using SPSS (version 23.0; IBM, NY, USA). Continuous data were analyzed using the t-test. Frequency data were analyzed using the chi-squared test (χ^2^). A correlation and logistic regression analysis was conducted to explore the interfering factors of Ct value fluctuation. Here, p < 0.050 was considered statistically significant.

## 3 Results

### 3.1 Overview of patients in two groups

A total of 192 patients were enrolled in this study; 95 of these patients had nucleic acid Ct value fluctuation (group A), and the 97 remaining patients had a normal discharge process and were regarded as the control (group B). There were no significant differences between the groups in age (t = 0.560, p = 0.576), gender distribution (χ2 = 0.017, p = 0.897), number of vaccination **dose** (t = −0.014, p = 0.989), initial ORF-Ct value (t = 0.712, p = 0.477), and initial N-Ct value (t = 0.587, p = 0.588). In group A, over 10% (10/95) of the patients were complicated with chronic internal disorders, such as hypertension, diabetes mellitus, and coronary disease; 35% (34/95) suffered from respiratory symptoms; and nearly 25% (23/95) had significantly abnormal chest radiology. The proportion of patients complicated with chronic internal disorders, respiratory symptoms, and abnormal chest radiology in group A was significantly higher than that in group B (all p < 0.05; **Table 1**).

**Table 1:** Overview of 192 patients in two groups.

Group	Number	Sex		age(years old)	Number of vaccination dose	Complicated with chronic internal disorders		Respiratory symptoms		Chest radiology		Initial ORF-Ct value	Initial N-Ct value
		male	female			Yes	No	Yes	No	Normal	Abnormal		
A	95	52	43	48.9 ± 14.9	1.9 ± 1.3	10	85	34	61	74	21	25.1 ± 4.8	24.3 ± 4.9
B	97	54	43	47.7 ± 13.8	1.9 ± 1.2	0	97	21	76	97	0	24.7 ± 4.4	23.9 ± 4.4
t/χ^2^		0.017		0.560	-0.014	8.745		4.695		24.075		0.712	0.587
P		0.897		0.576	0.989	0.003		0.030		<0.001		0.477	0.558

### 3.2 Comparison of Ct values of two groups

**Figure 1:**
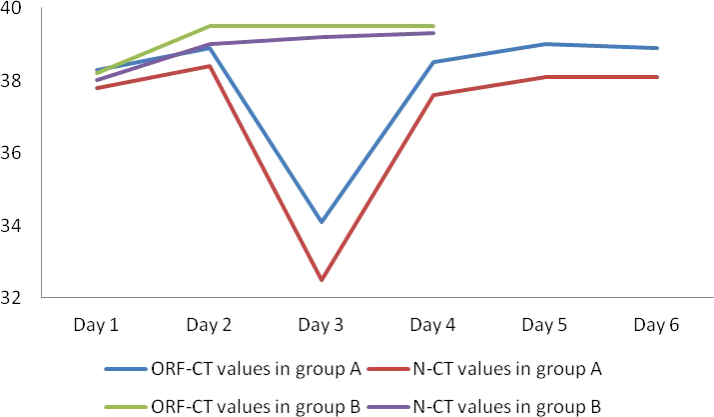
Nucleic acid Ct value fluctuation in two groups.

Between the two groups, there was no significant difference in the ORF-Ct or N-Ct value on day 1 (ORF: t = 0.316, p = 0.752; N: t = −0.583, p = 0.560). By contrast, the ORF-Ct and N-Ct values of group B on day 2 were significantly higher than those of group A (ORF: t = −2.962, p = 0.003; N: t = −2.276, p = 0.024). On days 3 and 4, the ORF-Ct and N-Ct values of group B were also significantly higher than those of group A (all p < 0.001). The mean Ct values of group A were over 35 on days 5 and 6 (**Table 2**; **Figure 1**).

**Table 2:** Nucleic acid Ct value fluctuation in two groups.

Group	Number	Day 1		Day 2		Day 3		Day 4		Day 5		Day 6	
		ORF-Ct value(Ct≥ 35)	N-Ct value(Ct≥ 35)	ORF-Ct value(Ct≥35)	N-Ct value(Ct≥35)	ORF-Ct value	N-Ct value	ORF-Ct value	N-Ct value	ORF-Ct value	N-Ct value	ORF-Ct value	N-Ct value
A	95	38.3 ± 1.7	37.8 ± 1.7	38.9 ± 1.6	38.4 ± 1.8	34.1 ± 2.9	32.5 ± 2.5	38.5 ± 2.4	37.6 ± 3.0	39.0 ± 1.8	38.1 ± 2.6	38.9 ± 4.5	38.1 ± 4.9
B	97	38.2 ± 1.8	38.0 ± 1.8	39.5 ± 1.1	39.0 ± 1.5	39.5 ± 1.1	39.2 ± 1.4	39.5 ± 1.0	39.3 ± 1.3	Discharged			
t		0.316	-0.583	-2.962	-2.276	-17.133	-22.926	-4.132	-5.108	/			
P		0.752	0.560	0.003	0.024	<0.001	<0.001	<0.001	<0.001	/			

### 3.3 Patient outcomes

No significant difference was observed between the groups in the ORF-Ct value at discharge (t = −0.639, p = 0.523), but there was a significant difference in the N-Ct value at discharge (t = −2.321, p = 0.022). The proportion of patients with negative conversion at discharge of group B was 93.8% (91/97), which was evidently higher than that of group A (χ^2^ = 4.554, p = 0.033). Seven days after discharge, almost 100% of the patients had been cured. The mean negative conversion interval of the patients in group A was 14.5 ± 4.6 days, which was longer than that of the patients in group B (11.8 ± 4 days; t = −4.314, p < 0.001) **(** **Table 3** **)**.

**Table 3:** Outcomes at discharge.

Group	Number	Discharge		negative converting at discharge(Ct ≥ 40)		negative converting 7days after discharge(Ct ≥ 40)		negative conversion interval of nucleic acid(days)
		ORF-Ct value	N-Ct value	No (%)	Yes(%)	No(%)	Yes(%)	
A	95	39.9±0.5	39.5±1.2	15(15.8%)	80(84.2%)	1(1.1%)	94 (98.9)	14.5±4.6
B	97	39.9±0.5	39.8±0.7	6 (6.2%)	91(93.8%)	0	97(100%)	11.8±4.0
t/χ^2^		-0.639	-2.321	4.554		0.001		-4.314
P		0.523	0.022	0.033		0.992		<0.001

### 3.4 Correlation–regression analysis of Ct value fluctuation

A correlation test was conducted to determine the association between Ct value fluctuation and the patients’ clinical characteristics and Ct values. There was a significant positive correlation between Ct value fluctuation and complication with chronic internal disorders, respiratory symptoms, and abnormal chest radiology. On the contrary, a significant negative correlation was identified between Ct value fluctuation and ORF-Ct and N-Ct values on day 2 (**Table 4**).

**Table 4:** Correlation the regression analysis of nucleic acid Ct value fluctuation.

	Complicated with chronic internal disorders	Respiratory symptoms	Abnormal chest radiology	ORF-Ct value on day 2	N-Ct value on day 2
r	0.237	0.156	0.373	-0.166	-0.181
P	0.001	0.03	<0.001	0.021	0.012

Logistic regression analysis was further conducted to explore the interfering factors of Ct value fluctuation. Findings showed that the ORF-Ct value on day 2 was the key factor impacting the fluctuation of Ct values (**Table 5**). The corresponding logistic regression equation is as follows:

**Table 5:** Regression analysis results of nucleic acid Ct value fluCtuation.

Variables in the equation	B	SB	Wald	P
X_1_: ORF-Ct value on day 2	-0.264	0.119	4.888	0.027
X_2_: Complicated with chronic internal disorders	20.701	11078.98	<0.001	0.999
X_3_: Abnormal chest radiology	21.183	7874.723	<0.001	0.998
constant	9.984	4.693	4.526	0.033

## 4 Discussion

### 4.1 Meaning of Ct value in China

The main pathogen of the current COVID-19 outbreak in Shanghai is the SARS-CoV-2 Omicron variant. Given the very small size of such organisms, the gold standard for virus detection is virus culture. However, due to the particularity of virus sampling and detection, the tested audience has an extremely limitation to meet with testing needs of over six hundred thousand patients in large-scale epidemics. Quantitative real-time reverse transcription polymerase chain reaction (RT-PCR) analysis of viral RNA from nasopharyngeal (NP) swabs is a common diagnostic method for SARS-CoV-2 (Baccini et al., 2021). The Ct value is the number of cycles that a target gene needs to be amplified in order to exceed the positive threshold (Tom and Mina, 2020). The use of the Ct value has been incorporated into Chinese guidelines for its rapid, high-volume testing mode.

Moreover, the Ct value has a certain correlation with the progression of COVID-19 (Rao et al., 2020; Abdulrahman et al., 2021). In 2020, a systematic review by Sonia N. Rao, which identified 11 studies on the COVID-19 Ct value, found a relationship between the Ct value and disease severity (Rao et al., 2020). Moreover, a 2021 study involving 192 patients showed a significant correlation between low Ct values and high mortality and shock (Samavedam et al., 2021). However, two studies in 2022 showed conflicting opinions about the limitations of reliably predicting the severity of COVID-19 (Penney et al., 2022; Shoaib et al., 2022).

The SARS-CoV-2 Omicron variant mainly invades the upper respiratory tract. Most hosts are asymptomatic or have mild respiratory symptoms. A small number of patients have pulmonary inflammation, and a few patients have severe symptoms. The present situation completely differs from that in 2020, where SARS-CoV-2 rapidly invaded the lungs and resulted in high mortality in Wuhan. This may be an important reason for the lack of a significant correlation between the Ct value of the Omicron variant and disease progression in this stage. On the contrary, for most asymptomatic patients, chest Ct and clinical symptoms cannot express disease outcomes. Therefore, in theory, the Ct value is an important indicator of disease outcomes, but there has been no study on the follow-up Ct values of patients who have met the discharge criteria. Such fluctuation in the Ct value motivates us to fill this research gap.

### 4.2 Reason for Ct value fluctuation

Different countries and health organizations recommend various Ct values (ranging from 25 to <40) as indicators of a positive result (Sule and Oluwayelu, 2020; Vogels et al., 2020). Although Ct values are quantitatively evaluated for the genomic number, which is influenced by many factors, including sample collection methods and heterogeneity of the environment (Lee et al., 2021), NP swabs are still considered the gold standard for evaluating infection and viral load with inversely correlated Ct values (Bullard et al., 2020; Sharfstein et al., 2020). In addition, daily sampling minimized the impact of swab procedures in our study, thus avoiding the possibility of underestimating a patient’s viral load.

In the process of patients infected with COVID-19, COVID-19 has fragment shedding phenomenon. In the process of disease recovery, small pieces of virus still fall off from respiratory mucosa irregularly. Once the above virus fragments are touched during sampling, the Ct value may fluctuate. As the research results, there were differences between the two groups of patients in the follow-up Ct values. In addition, since this fragment does not have obvious infectivity, the Ct value can quickly rise to more than 35 after 1-2 days. Therefore, we believe that the fluctuation of Ct value is only a normal phenomenon in the recovery period of the disease, and there is no need for excessive intervention.

In general, the envelope (ENV), nucleocapsid (N), spike (S), RNA-dependent RNA polymerase (RdRp), and ORF1 viral RNA genes are amplified by RT-PCR. Two highly conserved sequences, ORF1b and N, are selected to design primers and probes in China (Giri et al., 2021). As preliminary results of continuous dilution RNA samples showed that N gene analysis is about 10 times more sensitive than ORF1b gene analysis in detecting clinically positive specimens, Chu et al. recommended N gene screening and ORF1b as a confirmatory test (Chu et al., 2020). These clinical samples may contain infected cells expressing subgenomic mRNA during the initial course of the disease, resulting in increased copies of the N gene in the samples. In contrast, ORF was found to be correlated with Ct value fluctuation in the present study. As ORF1ab induces subdominant T cell responses and has a potential role in limiting disease severity (Swaminathan et al., 2022), monitoring ORF-Ct values may be more suggestive of asymptomatic patients.

Based on the above reasons, just because of the instability of the N gene segment, we found that there was a certain difference in the N-Ct value at discharge. However, from the numerical point of view, the mean N-Ct values between the two groups were close (A: 39.5 ± 1.2 *vs* B: 39.8 ± 0.7).

### 4.3 Appropriateness of setting of discharge Ct value to 35

According to Chinese guidelines, the nucleic acid Ct standard for discharge is 35. Our findings show that the nucleic acid results of most patients became negative when they were discharged from the hospital. According to the relevant policy of Shanghai, the nucleic acid should be reviewed one week after discharge. Through our follow-up, we found that almost all patients were negative during nucleic acid re-examination. Thus, despite the Ct value fluctuation, most patients turned out to be cured within a week of discharge.

In a study published in 2022, Ruian Ke et al. performed antigen detection and virus isolation and culture on 60 subjects for 14 consecutive days (Ke et al., 2022). Results showed that in most of the cases, the viral load increased and decreased dynamically in nasal swabs or saliva samples (Ke et al., 2022). Twelve days after onset, the viral Ct values of the nasal swabs and saliva samples of most patients were between 30 and 40 (Ke et al., 2022). The virus could not be isolated during the virus isolation and culture of all samples from the ninth day, indicating that the virus was no longer infectious (Ke et al., 2022). In a study assessing the infectivity of the virus, no infectious virus was isolated from the sample when the Ct value was greater than 35 (Ke et al., 2022). Therefore, it is reasonable to set the Ct value of the discharge standard to 35 in mobile cabin hospitals. Moreover, even if the Ct values of hospitalized patients fluctuate after two negative nucleic acid results, they can still be discharged without excessive intervention.

Nonetheless, this study found a certain correlation between Ct value fluctuation and the presence of comorbidities, respiratory symptoms, and chest imaging abnormalities. Judging from the number of days of the disease course and disease outcomes after follow-up, the average number of days of outcome of the patients in group A was higher by 2.7 days compared with that of the patients in group B. Therefore, different patients should be managed by stratification. If a patient has no underlying diseases and no symptoms of COVID-19, then the original plan can be maintained, and the nucleic acid can be reviewed seven days after discharge. On the contrary, if a patient has underlying diseases or symptoms related to COVID-19, the review time after discharge should be extended; that is, the nucleic acid should be retested on the tenth day after discharge, so as to avoid nucleic acid abnormalities due to virus shedding.

## Data availability statement

The raw data supporting the conclusions of this article will be made available by the authors, without undue reservation.

## Ethics statement

Written informed consent was obtained from the individual(s) for the publication of any potentially identifiable images or data included in this article.

## Author contributions

All authors contributed to the study conception and design. Material preparation, data collection and analysis were performed by XZ, SW, WZ, QS and MC. The first draft of the manuscript was written by XZ and YZ. All authors commented on previous versions of the manuscript. All authors contributed to the article and approved the submitted version.
